# Ultra-sensitive Magnetic Microscopy with an Optically Pumped Magnetometer

**DOI:** 10.1038/srep24773

**Published:** 2016-04-22

**Authors:** Young Jin Kim, Igor Savukov

**Affiliations:** 1 P-21, Los Alamos National Laboratory, P.O. Box 1663, MS-D454, Los Alamos, NM 87545, USA

## Abstract

Optically pumped magnetometers (OPMs) based on lasers and alkali-metal vapor cells are currently the most sensitive non-cryogenic magnetic field sensors. Many applications in neuroscience and other fields require high-resolution, high-sensitivity magnetic microscopic measurements. In order to meet this demand we combined a cm-size spin-exchange relaxation-free (SERF) OPM and flux guides (FGs) to realize an ultra-sensitive FG-OPM magnetic microscope. The FGs serve to transmit the target magnetic flux to the OPM thus improving both the resolution and sensitivity to small magnetic objects. We investigated the performance of the FG-OPM device using experimental and numerical methods, and demonstrated that an optimized device can achieve a unique combination of high resolution (80 *μ*m) and high sensitivity (8.1 pT/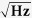). In addition, we also performed numerical calculations of the magnetic field distribution in the FGs to estimate the magnetic noise originating from the domain fluctuations in the material of the FGs. We anticipate many applications of the FG-OPM device such as the detection of micro-biological magnetic fields; the detection of magnetic nano-particles; and non-destructive testing. From our theoretical estimate, an FG-OPM could detect the magnetic field of a single neuron, which would be an important milestone in neuroscience.

All present magnetic field sensors involve a trade-off between resolution and sensitivity^1^. Currently, the most sensitive sensors are superconducting quantum interference devices (SQUIDs) and optically pumped magnetometers (OPMs)^2^,^3^, both with sensitivities below 1 f T/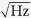, when the sensor size is on the order of a centimeter. Specially configured SQUIDs and Hall probes have micrometer scale resolution, however, this is achieved with sensitivity on the order of nT/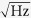. Such sensitivity limits their utility in recording magnetic fields of one or a small number of neurons, which would be a valuable experimental technique for studying brain function. Single neurons have dimensions of order of 10–100 *μ*m and are thought to produce ~10 pT magnetic fields at a 100 *μ*m stand-off^4^, which is out of reach of current technologies. Nitrogen-vacancy (NV)-diamond magnetometers, which can detect a single electron spin^5^, are promising, but they require irradiation by microwave power and that the target be placed in very close proximity to the NV centers, which is not always easy to achieve. Giant magnetoresistance and similar solid-state devices are also capable of high resolution, but the sensitivity is not sufficient for single neuron detection. A magnetic tunnel junction (MTJ) achieved a sensitivity of 133 pT/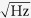 at 1 Hz^6^, but this required flux concentrators of the size of about 1 mm, limiting the resolution to the same order. Because of the limitations of these techniques, magnetic field measurements of brain activity, i.e. conventional magnetoencephalography (MEG), are performed with cm-scale sensors which measure the average field over 10^4^ to 10^5^ neurons. As a result, many important problems which require microscopic resolution remain unsolved. High-resolution, ultra-sensitive magnetometry, capable of detecting a single or a small number of neurons, would greatly aid in improving understanding of brain function and investigating the origins of the MEG signal at a small scale.

One way to improve resolution while maintaining high sensitivity is by miniaturizing OPMs^7^ operating in the spin-exchange relaxation-free (SERF) regime. Unfortunately the sensitivity-resolution tradeoff is far from optimal. When the OPM cell dimension is below 1 mm, spin relaxation is dominated by the spin-destructive collisions on the cell walls and *T*_2_ ~ *a*^2^ assuming a high buffer gas pressure within the cell^8^,^9^, where *T*_2_ is the transverse relaxation time and *a* is the cell dimension. (Note that in the case of the cell with no buffer gas, *T*_2_ ~ *a* due to ballistic motion of alkali-metal atoms^9^. The relaxation time in the evacuated cell would be much smaller than that in the cell with buffer gas unless a special antirelaxation coating is applied to the cell walls. The coating would impose constraints on the operation temperature and might be problematic in very small cells, so it is not clear if the coating strategy is better than the buffer gas technique.) The sensitivity is then determined both by spin-fluctuation noise, which goes as 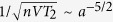, and by photon-shot noise, which goes as 1/*nlT*_2_ ~ *a*^−3^, where *n* is the density, *V* is the active volume, and *l* is the path length. Such miniaturization issues are exemplified by the 70 f T/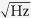 sensitivity obtained with a micro-fabricated mm-size OPM^7^, a sensitivity about 100 times worse than that of cm-size OPMs^3^, 0.5 f T/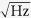, in qualitative agreement with the reported scaling. In addition, the finite thickness of the cell and heat insulating material, not to mention optical design constraints, can substantially increase the stand-off distance for smaller cells, making the miniaturization approach even less effective.

However, the above-mentioned difficulties in miniaturizing an OPM can be overcome with the approach that is the subject of this paper: combining a 1-cm size OPM with high permeability flux guides (FGs). Due to the inverse quadratic scaling of the sensitivity arising from the magnetic flux conservation in FGs, this approach is a more effective solution for reaching extremely small resolution than the miniaturization of the OPM since the ultimate resolution is determined by the size of FGs tips. With improved microfabrication techniques an FG-OPM could reach atomic-scale resolution. In addition, a 1-cm size OPM is now commercially available, making this approach suitable for fast commercialization. In terms of sensitivity, the performance will be limited both by the magnetic noise of FGs at low frequencies and the sensitivity of the OPM at high frequencies, as will be detailed later. This approach is similar to previously developed scanning SQUID microscopes, where high temperature superconductor SQUIDs were combined with a flux guide^10^,^11^. While they have considerably improved resolution down to a few tens of *μ*m for samples at room temperature, they achieved sensitivity of nT/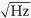 or less.

## Results

### Construction of FG-OPM probe microscope

**Figure 1:**
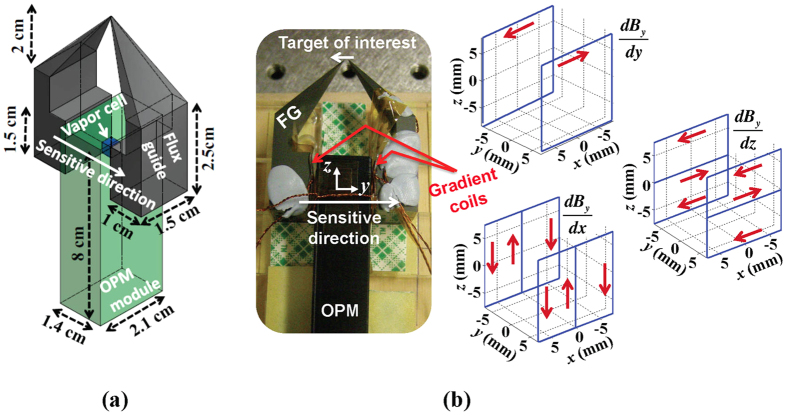
FG-OPM probe microscopy. (**a**) Schematic diagram of an FG-OPM microscope consisting of ferrite flux guides and a cm-scale optically pumped magnetometer. The OPM’s 3 × 3 × 3 mm^3^ Rb vapor cell is located in the center of the larger gap of the FGs. (**b**) Photograph of an FG-OPM. A magnetic target of interest is positioned near the probe tips and its magnetic flux is guided to the vapor cell in the *y* direction. Three gradient coils mounted in the larger gap of the FGs serve to compensate for gradients.

For proof-of-principle demonstrations, we constructed the FG-OPM probe microscope shown in Fig. 1. In order to avoid large magnetic Johnson noise, FGs were made of an electrically poorly conductive, high permeability (6500 at 10 k Hz) MnZn (MN60) ferrite, provided by Ceramic Magnetics Inc. A cm-scale SERF OPM containing a 3 × 3 × 3 mm^3^ Rb vapor cell was purchased from QuSpin Inc.^12^. The vapor cell, which defines the sensing volume, was located relative to the FGs as shown in Fig. 1. The magnetic targets were placed near the probe tips, so that magnetic flux from the target was transmitted towards the OPM vapor cell through the FGs, and the *y* component of the target’s field was measured. Because the magnetometer operates in the SERF regime in which there is no DC field across the cell, no flux is transmitted from the cell to the target.

### Investigation of FG-OPM sensitivity

The sensitivity of the FG-OPM was investigated inside a cylindrical ferrite shield with end-caps (18 cm diameter and 38 cm tall) inserted into a three-layer open mu-metal co-axial cylindrical shield (23 cm inner diameter, 29 cm outer diameter, and 69 cm tall). This combined shield provided sufficient suppression of the external DC field and magnetic noise, but the residual field inside the ferrite shield was compensated with three orthogonal coils and a complete set of five first-order gradient coils positioned inside the shield. For the FG-OPM sensitivity tests, several coils in the OPM gap of the FGs, Fig. 1b, were added to compensate for the residual field and gradients created by the FGs. In particular, the dominant *dB*_*y*_/*dz* gradient was suppressed by a factor of 100 with the *dB*_*y*_/*dz* gradient coil. A square Helmholtz coil with 2.5 cm sides was mounted near the vapor cell to generate a calibration field in the *y* direction at 80 Hz in order to convert the OPM output voltage spectrum into the magnetic field spectrum.

**Figure 2:**
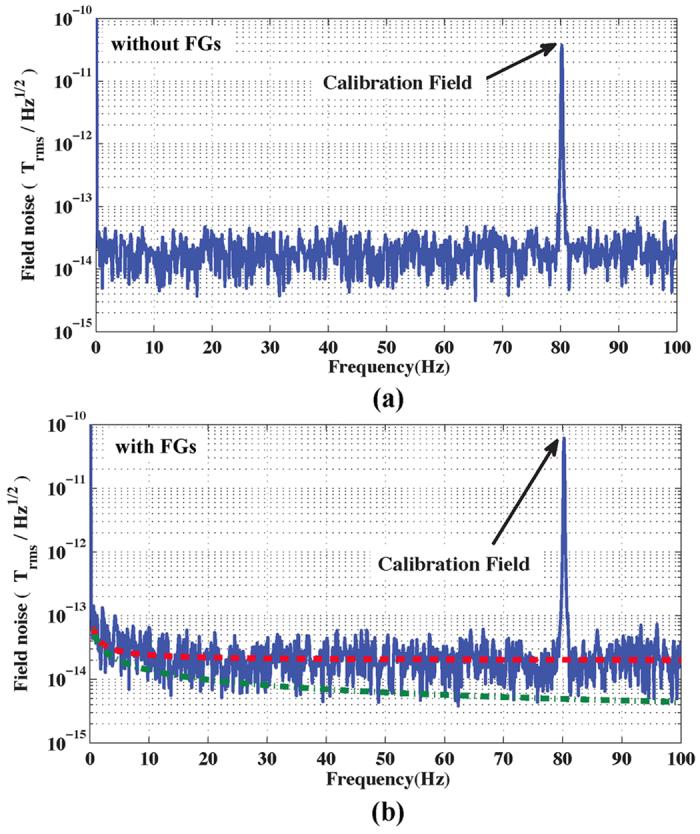
Intrinsic sensitivity measurements of the OPM and FG-OPM. The calibrated magnetic field noise of both the OPM without FGs (**a**) and the combined FG-OPM (**b**) was measured inside a cylindrical ferrite shield, inserted into a three-layer open mu-metal co-axial cylindrical shield, that included compensation coils to remove ambient DC fields. A uniform calibration field at 80 Hz was applied in the *y* direction to convert the measured voltages into magnetic fields. The 63% bigger calibration field strength in (**b**) is due to field enhancement by FGs. The calibrated noise spectrum takes this factor into account. The intrinsic sensitivity of the OPM and combined FG-OPM, averaged from 65 Hz to 78 Hz, was 19.7 f T/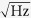 and 20.2 f T/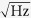, respectively. In (**b**), the dashed red line shows the estimated total magnetic noise of the FG-OPM and the dashed green line shows the estimated thermal magnetic noise originating from the FGs, based on Eq. 1 and the noise of the OPM.

Figure 2a shows the calibrated field noise spectrum of the OPM without FGs demonstrating its intrinsic sensitivity, averaged from 65 Hz to 78 Hz, of 19.7 f T/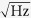. The bandwidth of the OPM was measured to be 137 Hz. Note that since our measurements, QuSpin has improved the sensitivity of its sensors to 10 f T/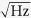 and we expect to improve the performance in the future by upgrading the first model.

In Fig. 2b we present the calibrated field noise spectrum of the combined FG-OPM. The OPM was tuned by adjusting all of the compensation and gradient coils described above to optimize the signal strength from a small oscillating calibration field^13^. The minimum separation between the probe tips was set at 50 *μ*m (the tip geometry discussed below). The intrinsic sensitivity of the FG-OPM was measured to be 20.2 f T/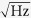 averaged from 65 Hz to 78 Hz, with a bandwidth of 147 Hz, performance which is very close to that of the OPM without FGs. The results imply that the addition of the FGs did not significantly impair the performance of the OPM once the residual field and gradients of the FGs were properly compensated.

**Figure 3:**
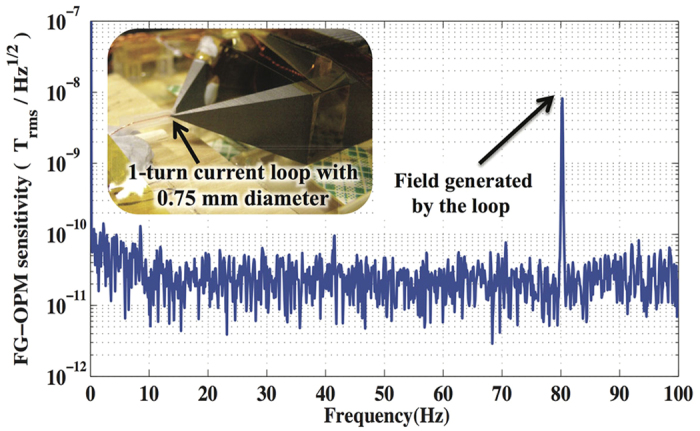
FG-OPM sensitivity to a small magnetic object near the probe tips. The magnetic field sensitivity of the FG-OPM to a magnetic target near the tips was measured with a 0.75 mm-diameter current loop placed between the tips. Knowing the absolute field strength generated by the loop at 80 Hz allowed us to calibrate the field spectrum of the FG-OPM, revealing that the sensitivity of the FG-OPM was 23 pT/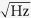.

To determine the FG-OPM sensitivity to a magnetic source located near the probe tips, which is the important characteristic of the microscope, a small 0.75 mm-diameter current loop was placed between the tips as shown in the inset of Fig. 3. A field generated by the loop was detected with the FG-OPM and the FG-OPM output spectrum was calibrated to the field strength of the calibration field. From the field generated by the loop and the calibrated field spectrum of FG-OPM, we found that the field transfer coefficient of the FGs, the ratio of the measured field to the generated field, was 8.7 × 10^−4^, which results in the sensitivity of 23 pT/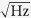 shown in Fig. 3.

### Three-dimensional numerical simulations

**Figure 4:**
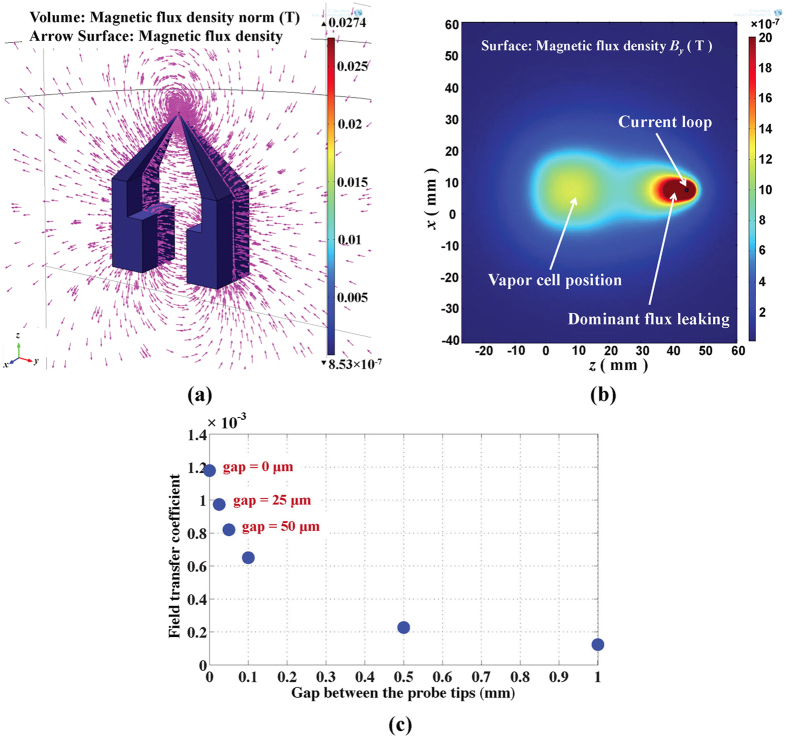
Three-dimensional numerical simulations. (**a**) The magnetic field distribution in the FGs visualized with field lines. (**b**) A two-dimensional projection of *B*_*y*_ onto the *xz* plane at the center of FGs indicates that the magnetic flux leaks out predominantly near the FG tips. (**c**) Calculated field transfer coefficient vs. gap between the probe tips. The calculation is in 5% agreement with the measurement for the tips gap of 50 *μ*m.

In order to examine how the FGs transmit the magnetic flux generated from the current loop and theoretically analyze the sensitivity, we performed several three-dimensional simulations using finite element analysis software (COMSOL Multiphysics 4.3). As indicated in Fig. 4a,b, simulations revealed that the magnetic flux leaked predominantly near the probe tips. This leads to a loss in flux and a reduction of the field detected by the OPM. Figure 4c shows the calculated field transfer coefficient as a function of the gap between the probe tips. The theoretical result of 8.3 × 10^−4^ agrees well with our experimental result of 8.7 × 10^−4^ for the tip gap of 50 *μ*m. Because the flux predominantly leaks out around the probe tips, the optimization of the tips would be necessary for further sensitivity improvement. The simulations also show that the field transfer coefficient can be improved by a factor of two when the separation between the FGs at the OPM end (currently about 1.4 cm) is decreased by a factor of two. This was not attempted with our current OPM since it would have required a redesign of the sensor head.

### Estimate of thermal magnetic noise from FGs

The thermal magnetic noise originating from the FGs was estimated using the equations derived from the fluctuation dissipation theorem^14^:


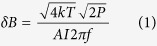


where *k* is Boltzmann’s constant, *T* is the absolute temperature, *P* is the power loss, and *f* is the frequency at which noise is considered. For the weakly conductive NM60 ferrite (*σ* = 0.2 Ω^−1^ m^−1^), the power loss is dominated by the hysteresis losses: 
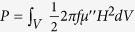
, where *μ*′ and *μ*′′ are the real and imaginary parts of the complex permeability *μ* = *μ*′ − *iμ*′′ and *H* is the magnetic field generated by a hypothetical excitation coil (1 turn, small area *A*, and current *I*) placed in the position of the noise measurement^14^. The integration is carried out over the total volume of the FGs using the simulated fields^14^,^15^. We adopted the value of *μ*′′ (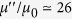) in^15^ because the same ferrite material (MN60) was used.

The magnetic noise from the FGs in the vapor cell location was estimated to be 44.2 *f*^−1/2^ f T/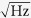 at 300 K. The predicted magnetic noise is 44 f T/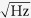 at 1 Hz and 5 f T/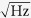 at 80 Hz. The dashed green and red lines in Fig. 2b show the estimated FG noise *δB*_FG_ and the total magnetic noise 
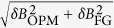
, where *δB*_OPM_ is the noise of the OPM. This means at low frequencies the noise is dominated by *δB*_FG_, while at high frequencies by *δB*_OPM_. Therefore, at low frequencies the sensitivity can be improved through better FG design or by cooling the ferrite material, while at high frequencies, improvement in sensitivity should focus on the OPM design.

### Investigation on resolution of FG-OPM

**Figure 5:**
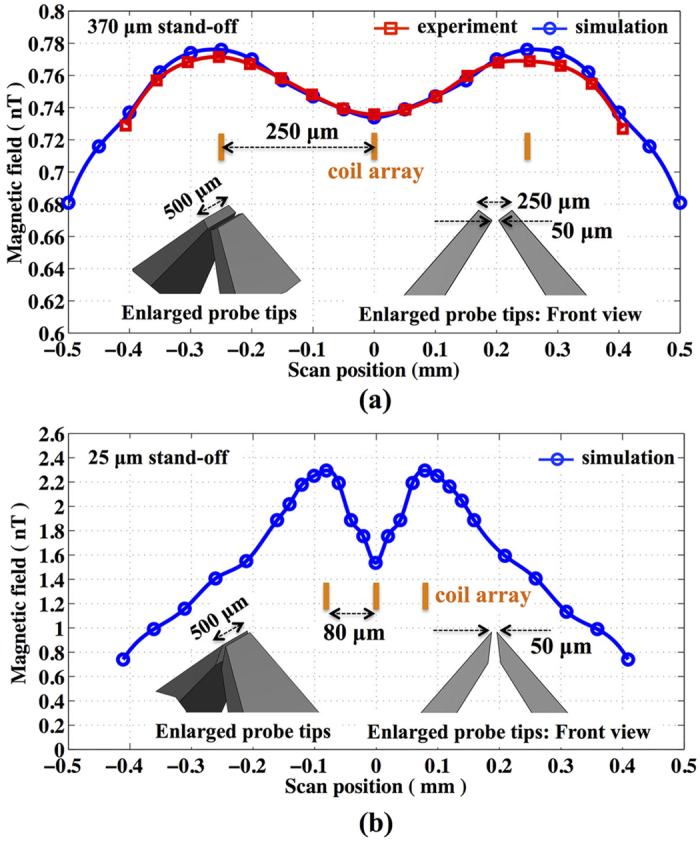
Investigation of the FG-OPM spatial resolution by displacement of an array of three 0.75 mm-diameter loops near the probe tips. (**a**) The experimentally measured and numerically calculated magnetic fields at the position of the vapor cell as a function of tip position obtained with a coil array configured with a separation of 250 *μ*m and anti-parallel currents. The measurement clearly detected two maxima and one minimum and shows the best resolution (250 *μ*m) that the FG-OPM of the manufactured FG tips shape could achieve. The resolution was limited by the upper tip gap of 250 *μ*m as shown in the inset. (**b**) Simulations reveal an improved resolution of 80 *μ*m using optimized FGs with sharper tips, shown in the inset, and a tip gap of 50 *μ*m. Two maxima and one minimum were clearly detected with the 80 *μ*m coil separation.

The resolution of the FG-OPM was investigated both experimentally and numerically by scanning the position of an array of three one-turn 0.75 mm-diameter coils at specific stand-off distances from the probe tips. In the experiment, a 80 *μ*m (40 gauge) wire was used for the coil array to carry the 56 mA current. The direction of the current in the middle coil was reversed. We tried to find the limit of resolution achievable with the manufactured FGs tips by varying the coil separation distances and then finding the conditions where the three coils were clearly discernible in the observed signal. The inset of Fig. 5a shows the enlarged FGs probe tips and their specific dimensions. Due to the brittle nature of the ferrite, these tips were the best we could manufacture. The stand-off distance was difficult to measure directly, but was determined to be 370 *μ*m by comparing the data to the simulations. The measurements and simulations, which clearly observed two maxima and one minimum for the coil separation of 250 *μ*m, shown in Fig. 5a showed that the best resolution was 250 *μ*m and that it was limited by the upper tip gap. Since the simulation reproduced the experimental results, further analysis of resolution and sensitivity with optimized FGs was conducted solely through numerical simulations.

In order to investigate the significance of the tip geometry, the model of the probe tips was modified as shown in Fig. 5b, with the upper tips made sharper and the gap reduced to 50 *μ*m. With the stand-off of the coil array at 25 *μ*m, the wire diameter set to 40 *μ*m, and the direction of the 56 mA current in the middle coil reversed, we found that the resolution for this configuration was better than 80 *μ*m. As shown in Fig. 5b, two maxima and one minimum can clearly be observed for a 80 *μ*m coil separation. Our expected field transfer coefficient for the newly designed FGs is 1.23 × 10^−3^, 40% better than that of the FGs we experimentally tested. With the improved OPM from QuSpin operating at 10 f T/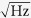 sensitivity, we estimate the sensitivity of the new FG-OPM to a magnetic source of interest located at the probe tips would improve to 8.1 pT/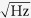. Thus an optimized FG-OPM is expected to reach a resolution of 80 *μ*m and sensitivity 8.1 pT/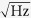, which could be sufficient to detect the magnetic field of a single neuron after about 10 to 1000 averages, depending on some specific parameters of neurons. The recovery time of neurons after a stimulation will be a determining factor in a choice of the measurement repetition time.

## Discussion

An optimized FG-OPM has sufficient resolution and sensitivity for the detection of a single or a small number of neurons or functional domains of 0.3–0.6 mm size, in which neurons show a coherent response. This is one of many possible applications of the FG-OPM to help understand the structure and function of the brain at different scales. The micro-imaging magnetic measurements will be valuable for neurosurgical planning; establishing the neurological basis of epilepsy, Alzheimer’s, and stroke; developing diagnostic methods, drugs, and treatments; and studying cognitive and perceptual responses^16^. Microscopic resolution is important because the dynamics observed at the macroscopic scale originates from the microscopic level^17^. In addition to studies of human brain functions, the FG-OPM can be used to record MEG signals in small animals, which are widely used in neuroscience research because studies of fundamental brain function can be done in correlation with other invasive methods. Animals are also essential for studies of diseases and treatments, for example, for drug evaluation before human trials where brain response can be studied directly. The relatively small brain size in these subjects increases the requirement for high resolution and sensitivity. Microscopic measurements with the FG-OPM require very close approach to objects, which is going to be invasive. This is feasible in open- or thin-skull measurements. A competing technology of invasive methods is microelectrodes inserted into brains, which directly records change in voltage potential within cells^18^. Compared to the implanted electrodes, the benefit of the FG-OPM is less invasive non-contact measurement that will avoid several problems in the contact measurement such as disturbance of neurons^16^ and the immune reaction^19^.

Beyond neuroscience, an FG-OPM can be applied to magnetic nano-particle detection, which are proving to be an important and versatile probe in fields ranging from medicine and biophysics to oil exploration. For example, targeted nano-particles in cancerous tissues can be used to optimize cancer detection sensitivity^20^. The FG-OPM’s advantage of a small stand-off distance is especially important in *ex vivo* applications in bioassay, where an FG-OPM can monitor nano-particles (typically tagged to cells) in solution flowing less than 100 *μ*m from the probe tips of the FG-OPM. Decreasing the stand-off distance produces enormous gain in sensitivity – the nano-particle dipole field falls off as the cube of the distance – and we estimated that an FG-OPM should detect a single magnetic nano-particle as small as 30 nm after a few seconds of measurement time. Finally, FG-OPM resolution and sensitivity are expected to be sufficient for non-destructive testing (NDT), in which defects using either natural magnetic properties of materials or induced magnetic fields by applied currents, AC magnetic fields (eddy currents), and other methods can be revealed and studied. For example, counterfeit integrated circuits, which adversely affect system performance and lives, have become a major challenge over the last few decades. An FG-OPM can be used to authenticate integrated circuits and detect counterfeit and tampered parts. Superconducting quantum interference devices have been at the forefront of NDT applications due to their outstanding combination of sensitivity and resolution^21^. However, an FG-OPM would successfully compete with SQUID technology, with the advantages of non-cryogenic operation and decreased stand-off distances.

The large size of FG-OPM precludes manufacturing of an FG-OPM array for simultaneous measurements. Instead, this is analogous to the wide range of scanning probe microscopy technologies. In order to accelerate scanning, it would be realizable to combine a few FG-OPMs.

In conclusion, we have constructed an FG-OPM probe microscope and demonstrated that it has the combined resolution of 250 *μ*m and sensitivity of 23 pT/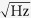, limited by the probe tip geometry. The numerical simulations show that optimized FGs can improve the resolution to 80 *μ*m with a sensitivity to 8.1 pT/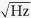, when using an OPM with 10 f T/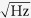 sensitivity, now commercially available from QuSpin.

## Methods

### Optically Pumped Magnetometer

A SERF cm-scale OPM manufactured by QuSpin Inc. was employed for the experiments. It contains a 3 × 3 × 3 mm^3 87^Rb vapor cell and a single laser for optical pumping and probing. The OPM was easily operated using a control software provided by QuSpin. The cell was heated to around 160° and a 926 Hz modulation field was applied in the *y* direction, required for the parallel probe-pump beam configuration.

## Additional Information

**How to cite this article**: Kim, Y. J. and Savukov, I. Ultra-sensitive Magnetic Microscopy with an Optically Pumped Magnetometer. *Sci. Rep.* **6**, 24773; doi: 10.1038/srep24773 (2016).
